# Glucocortiocoid Treatment of MCMV Infected Newborn Mice Attenuates CNS Inflammation and Limits Deficits in Cerebellar Development

**DOI:** 10.1371/journal.ppat.1003200

**Published:** 2013-03-07

**Authors:** Kate Kosmac, Glenn R. Bantug, Ester P. Pugel, Djurdjica Cekinovic, Stipan Jonjic, William J. Britt

**Affiliations:** 1 Department of Microbiology, University of Alabama at Birmingham, Birmingham, Alabama, United States of America; 2 Department of Pediatrics, University of Alabama at Birmingham, Birmingham, Alabama, United States of America; 3 Department of Histology and Embryology, Faculty of Medicine University of Rijeka, Rijeka, Croatia; 4 Department of Neurobiology, University of Alabama at Birmingham, Birmingham, Alabama, United States of America; University of California, San Diego, United States of America

## Abstract

Infection of the developing fetus with human cytomegalovirus (HCMV) is a major cause of central nervous system disease in infants and children; however, mechanism(s) of disease associated with this intrauterine infection remain poorly understood. Utilizing a mouse model of HCMV infection of the developing CNS, we have shown that peripheral inoculation of newborn mice with murine CMV (MCMV) results in CNS infection and developmental abnormalities that recapitulate key features of the human infection. In this model, animals exhibit decreased granule neuron precursor cell (GNPC) proliferation and altered morphogenesis of the cerebellar cortex. Deficits in cerebellar cortical development are symmetric and global even though infection of the CNS results in a non-necrotizing encephalitis characterized by widely scattered foci of virus-infected cells with mononuclear cell infiltrates. These findings suggested that inflammation induced by MCMV infection could underlie deficits in CNS development. We investigated the contribution of host inflammatory responses to abnormal cerebellar development by modulating inflammatory responses in infected mice with glucocorticoids. Treatment of infected animals with glucocorticoids decreased activation of CNS mononuclear cells and expression of inflammatory cytokines (TNF-α, IFN-β and IFNγ) in the CNS while minimally impacting CNS virus replication. Glucocorticoid treatment also limited morphogenic abnormalities and normalized the expression of developmentally regulated genes within the cerebellum. Importantly, GNPC proliferation deficits were normalized in MCMV infected mice following glucocorticoid treatment. Our findings argue that host inflammatory responses to MCMV infection contribute to deficits in CNS development in MCMV infected mice and suggest that similar mechanisms of disease could be responsible for the abnormal CNS development in human infants infected in-utero with HCMV.

## Author Summary

Intrauterine infection with human cytomegalovirus (HCMV) is a leading cause of developmental brain damage. In the U.S., an estimated 2,000 infants a year develop brain damage as a result of intrauterine infection with HCMV. In this study, we examined the contribution of host immune responses induced by CMV infection to abnormal development of the CNS by treating neonatal mice infected with MCMV with glucocorticoids. We found that glucocorticoid treatment of infected mice decreased the inflammatory response within the CNS without altering the level of virus replication. In addition, abnormalities in the structure of the cerebellum, as well as abnormalities in granule neuron precursor cell proliferation were normalized in MCMV infected mice following glucocorticoid treatment. These studies suggest that the host immune response to CMV infection is damaging to the developing CNS and that it may be possible to limit CNS disease by modulating inflammation. Moreover, understanding how inflammation and the immune response may alter the developmental program within the CNS could offer important insight into the mechanisms of disease leading to abnormal brain development following intrauterine infection.

## Introduction

Viral infections in the fetus and young infant are well described causes of abnormal brain development that often result in permanent neurological sequelae, including disorders of motor and cognitive functions. Altered CNS development and neurologic disease have been documented in the developing fetus and young infant following infection with a number of viruses, such as herpes simplex virus (HSV), rubella, lymphocytic choriomeningitis (LCMV) and human cytomegalovirus (HCMV) [1]–[7]. A variety of mechanisms can lead to interruption of the developmental program of the CNS including: damage to the brain parenchyma secondary to apoptotic or necrotic loss of resident cells within the CNS, damage to the supporting vasculature and microvascular supply of the CNS resulting in decreased blood flow and/or damage to the blood brain barrier, altered cellular positioning and disruption of synapse formation leading to a failure in neuronal connectivity and circuitry formation [8], [9]. In the case of infection with viruses that exhibit specific cellular tropism, the loss or dysfunction of specific populations of resident cells within the CNS often underlies disease. In other cases, cellular tropism is broad and disease is thought to result from direct viral damage to supporting structures, such as the vasculature or the glial architecture. Additionally, indirect mechanisms of disease following CNS infection include viral induced host inflammatory responses [10], [11]. Host responses following virus infections often lead to more global CNS damage secondary to the production of soluble effector molecules that can amplify proinflammatory responses of resident cells as well as promote cytotoxic activity by effector cells of the adaptive immune system [12]–[23]. Although these mechanisms of disease, as well as other proposed mechanisms, are consistent with clinical findings in patients with viral encephalitis, a precise description of the pathogenesis of CNS disease in virus infected human fetuses and infants is often limited by the lack of informative tissue specimens.

Because of limitations inherent in studies of the human CNS, small animal models have been developed to elucidate mechanisms of disease associated with viral infections of the developing CNS. These models have utilized a number of different viruses including HSV, murine cytomegalovirus (MCMV), LCMV, alphaviruses and more recently West Nile Virus (WNV) [4], [24]–[30]. Studies of CNS disease following both peripheral and intracerebral HSV inoculation have described a necrotizing encephalitis, which is more severe in animals with deficits in innate and adaptive immunity [31]–[33]. However, more recent studies have argued that in addition to the direct cytopathic effects associated with HSV replication, host derived innate immune responses contribute to CNS damage in infected mice [34], [35]. Similarly, experimental models employing LCMV infection have provided direct evidence that host-derived inflammation is a major component of CNS disease [4], [36]. In these models, limiting CD8+ virus specific T lymphocyte responses, or more global immunosuppression, dramatically reduced the severity of CNS disease [4], [37]. The contribution of immunopathological responses are particularly relevant to disease in young animals because expression of inflammatory genes during the dynamic developmental program of the CNS appears to result in a disease phenotype that differs from that seen in adult animals. Thus, substantial CNS damage in young infants could result from infection with viruses that are infrequently pathogenic in adults. In contrast, an effective immune response does appear to be necessary to limit the severity of CNS infection with alphaviruses and WNV [24]–[26], [38]–[42]. Responses derived from the adaptive immune system, in particular the production of antiviral antibodies, determine the susceptibility of newborn animals to alphavirus infection of the CNS [25], [38], [43], [44]. Thus disease outcome in young animals with viral infections of the CNS reflects a balance between unregulated inflammation and the control of virus replication [18], [32], [45]–[51].

Intrauterine infection with HCMV is the most common cause of congenital (present at birth) infection in humans and occurs in approximately 1/200 live births in the United States [52]. A small but significant number of newborn infants infected in-utero exhibit a variety of neurodevelopmental abnormalities secondary to HCMV infection of the CNS [5], [6]. Because little is known about the mechanisms of disease associated with this intrauterine infection, we developed a murine model of CNS infection that utilizes peripheral inoculation of newborn animals with limiting amounts of MCMV. In contrast to other murine models that have utilized intracranial inoculations of MCMV almost exclusively, the model we have developed uses intraperitoneal inoculation of limiting amounts of MCMV and requires virus replication in the periphery, viremia and neuroinvasion. These latter features of this murine model, particularly the hematogenous spread to the CNS, appear to more closely recapitulate the presumed pathogenesis of fetal CNS infection with human cytomegalovirus. MCMV infection of the brain in these newborn mice results in a focal, non-necrotizing encephalitis with little evidence of specific cellular tropism but with global and symmetric deficits in brain development [53]. Altered development occurred in areas of the brain that exhibited no evidence of viral proteins or nucleic acids, suggesting that inflammatory responses to infection, and not direct effects of virus infection, were responsible for the altered development in the brain of neonatal animals [53]. To determine the potential role of host derived inflammation as a mechanism of disease in this model, we first needed to separate the linkage between virus replication and host inflammatory responses. This was accomplished by treating young animals with corticosteroids to limit host responses, and therefore inflammation, during virus infection. Although inflammation in MCMV infected animals was reduced at several levels, viral replication was unaffected. More importantly, the anti-inflammatory activity of corticosteroids attenuated the previously described developmental abnormalities in the cerebella of infected animals. This finding strongly argued that virus replication was not a direct cause of the developmental abnormalities within the CNS following MCMV infection and suggested that inflammatory responses played a major role in the disease phenotype [53].

## Results

### Focal MCMV infection of the early postnatal CNS results in a robust inflammatory response within the CNS

**Figure 1:**
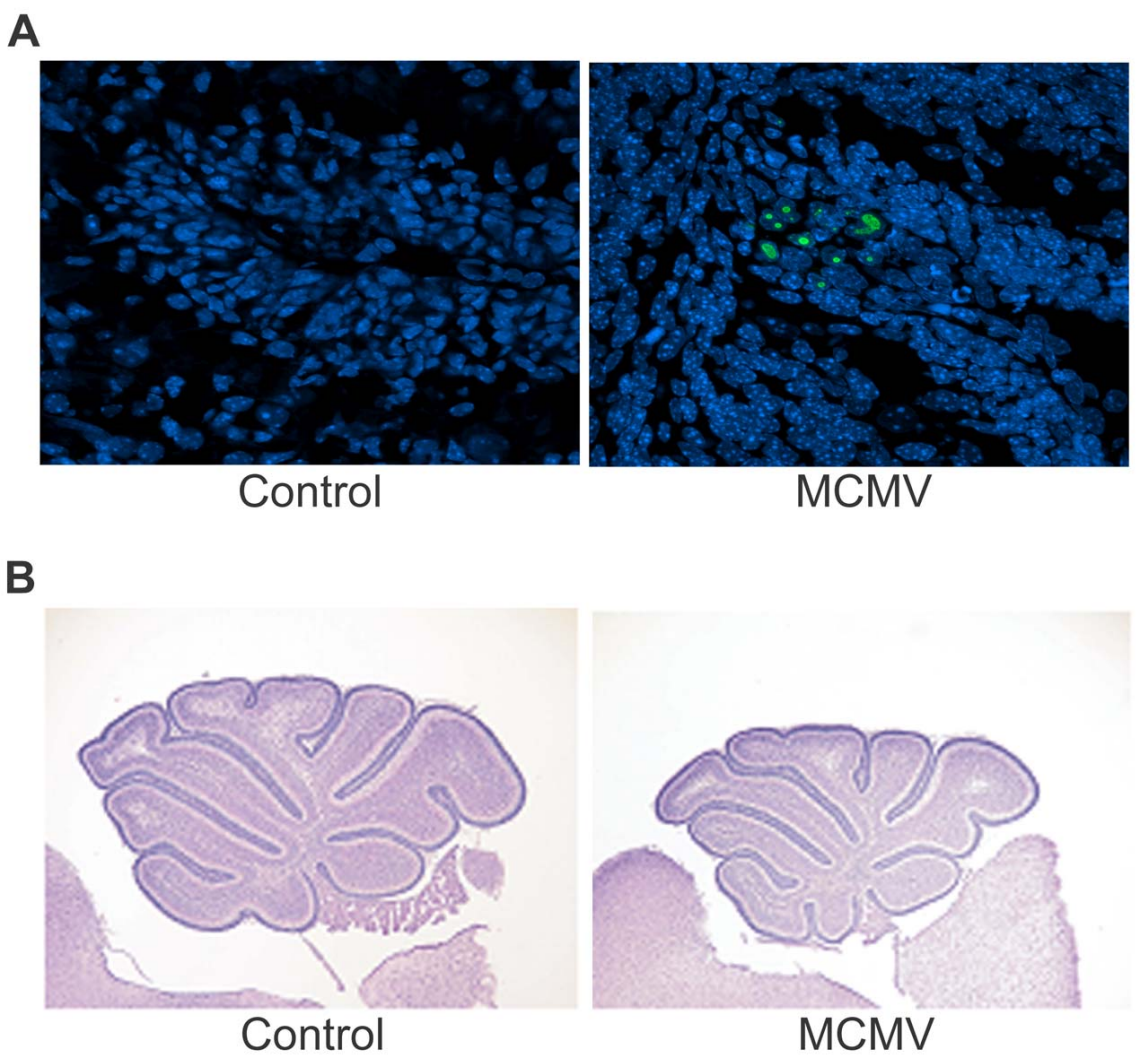
Neonatal infection with MCMV results in a focal encephalitis with global deficits within the cerebellum. A. Expression of immediate early gene 1, protein pp89 (IE-1) (green) in the cerebellum, a non-structural protein encoded by MCMV very early in infection, PND8, 60×. Note the focal nature of infection in the external granule cell layer (EGL) of the cerebellar cortex. B. Cresyl violet staining showing a global effect of virus infection on cerebellar area and folia development, 4×. Note the smaller size, delayed foliation and delayed fissure formation in the cerebellum of infected animals.

In an earlier report we described altered cerebellar development, including delayed cortical lamination, associated with MCMV infection of the CNS in newborn mice [53]. Disruption of lamination within the cerebellar cortex was frequently observed; however, altered lamination in areas immediately adjacent to virus infected cells was atypical in an overwhelming number of examined sections. Thus, histologic evidence of direct virus cytopathology as a cause of abnormal lamination of the cerebellar cortex was rare (Figure 1A). The predominant histopathologic findings of this CNS infection were widely distributed foci of virus infected cells and surrounding mononuclear cells throughout the cerebrum and cerebellum [53]. In contrast to the focal nature of virus infection and mononuclear cell infiltration, defects in cerebellar morphogenesis were global and, most importantly, symmetric as illustrated by the delayed foliation and reduced cerebellar area in virus infected animals (MCMV) compared to uninfected (control) animals at post-natal day (PND) 8 (Figure 1B). Notably, studies of infants infected in-utero by HCMV have also described global and symmetric deficits in brain morphogenesis without a significant component of focal or asymmetric loss of brain parenchyma, in the majority of documented cases [6], [54]–[61]. From these findings, we have proposed that global alterations in cerebellar development are likely associated with soluble factors produced by the host inflammatory response and not related to direct effects of viral cytopathology.

**Figure 2:**
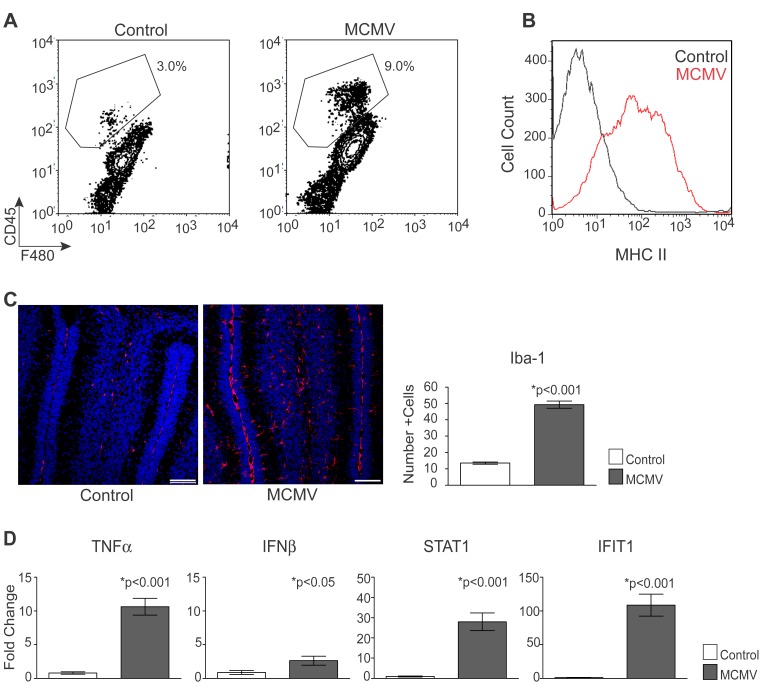
Infiltration of inflammatory cells and induction of proinflammatory cytokines in the brains of MCMV infected mice. A. Percentage of CD45^hi/int^, F4/80^+^ mononuclear cells in the brain following infection with MCMV, PND8. Plots are representative of 1 of 4 replicates, n = 4 mice pooled/replicate. B. Expression of MHC Class II, gated on CD45^hi/int^, F4/80^+^ population. Histogram is representative of 1 of 4 replicates, n = 4 mice pooled/replicate. C. Expression of Iba-1 (red), a marker for activated macrophages/microglia, and TOPROIII (blue), a nuclear marker, in the cerebellum of control and infected mice at PND8, 20×, scale bars = 50 µm. The number of Iba-1^+^ cells was quantified from 4 sections/animals, n = 8 mice/experimental group. Data are shown as mean +/− SEM. P values were calculated using a two-tailed T test. D. Inflammatory gene expression in the cerebellum of control and infected mice at PND8. Data are shown as mean +/− SEM. P values were calculated using a two-tailed T test, n = 5 mice/experimental group.

To characterize the nature of the inflammatory response in the cerebellum of infected animals, we analyzed several immunologic parameters in the brains of control and infected animals at PND8. This time point was selected because virus replication in the CNS was established and deficits in cerebellar development were clearly observable [53]. Initially, we assayed the phenotype of CNS mononuclear cells in control and virus infected animals. Although CD8^+^ and CD4^+^ T-lymphocyte infiltrates, peripheral blood macrophages and activated microglia could be readily detected in the cerebellar parenchyma at PND14, mononuclear cells were present in the CNS of MCMV infected mice by PND8, prior to the detection of infiltrating T-lymphocytes [62]. Mononuclear cells isolated from control and infected brains were stained with two markers for tissue macrophages, F4/80, a marker for cells of myeloid lineage and CD45, a pan-leukocyte marker. The differential expression of CD45 by F4/80^+^ cells has been employed to distinguish between quiescent microglia (low), activated microglia (intermediate) and infiltrating macrophages (hi) [63]. In control animals, F4/80^+^ cells expressing CD45^hi/int^ were present in low abundance (3.0%) (Figure 2A). We observed an increase in the proportion of CD45^hi/int^F4/80^+^ cells in the CNS of infected mice (9%) (Figure 2A) [62]. Furthermore, MHC class II expression was increased in this population of cells in MCMV infected mice, a finding consistent with the activation of these cells following infection (Figure 2B). These results demonstrated an increase in the inflammatory response within the CNS, including increased activation of resident macrophages and recruitment of peripheral blood macrophages early in infection, prior to the appearance of virus specific CD8^+^ T-lymphocytes. To further define the activation state of brain macrophages in the CNS of MCMV infected mice, cerebellar sections from PND8 control and infected animals were stained with anti-Iba-1, a marker for activated microglia/macrophages [64], [65]. In sections from the cerebella of control mice, few Iba-1^+^ cells were observed (Figure 2C). However, the number of Iba-1^+^ cells in the cerebellum was significantly increased following infection with MCMV (Figure 2C). In addition, Iba-1 staining was observed in the meningial layer within the cerebellum of MCMV infected animals, suggesting an infiltration of cells from the periphery (Figure 2C). Importantly, cellular infiltrates and activated mononuclear cells in the cerebellum were readily detected in the parenchyma of the cerebellum and not limited to foci of virus infected cells (data not shown), suggesting that the generalized inflammation observed in the brains of MCMV infected mice was induced by soluble mediators produced in response to virus infection. Finally, we attempted to determine the frequency of Iba-1^+^ cells with an ameboid morphology suggestive of activated microglia and/or macrophages as compared to Iba-1^+^ cells with a ramified morphology consistent with quiescent or resting microglia/macrophages. We found cells consistent with both morphologies in infected and control animals but were unable to definitively assign differences in populations between the two experimental groups (data not shown).

Given the increase in the number of Iba-1+ cells and the increased activation of CD45^hi/int^F4/80^+^ cells, we next quantified the expression of inflammatory cytokines in virus-infected cerebella by quantitative real time PCR. We selected several proinflammatory cytokines, as well components of interferon induced responses (IFIT1 and STAT1), as markers for inflammation in the cerebella of infected animals. The expression of TNFα (10-fold), IFNβ (7-fold), STAT1 (10-fold) and IFIT1 (175-fold) were significantly increased in infected animals as compared to control animals (Figure 2D). Together, these results demonstrated that by PND8 activated cells of the innate immune response and proinflammatory cytokines were present in the developing cerebellum of mice infected with MCMV as newborns.

### Treatment of virus-infected mice with corticosteroids decreases CNS inflammation

**Figure 3:**
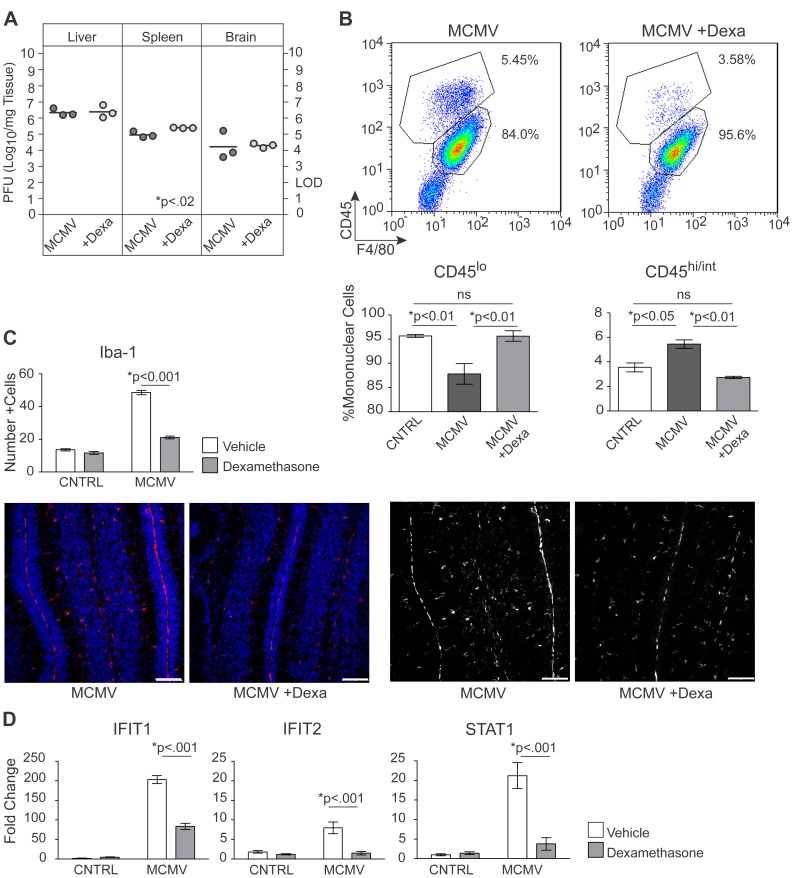
Treatment of MCMV infected neonates with dexamethasone decreases infiltration of inflammatory cells and expression of interferon stimulated genes in the CNS without increasing levels of virus replication. A. Infectivity assay showing viral titers in the liver, spleen and brain of infected mice treated with vehicle or dexamethasone (dexa). Each circle represents plaque forming units (PFU)/mg of tissue for an individual animal, p values calculated using two-tailed T test. B. (Top) Dot plots showing the percentage of CD45^lo^ and CD45^hi/int^, F4/80^+^ mononuclear cells in the brain of infected animals following treatment with dexa, gated on mononuclear cells. Plots are representative of 1 of 4 replicates. (Bottom) Bar graphs showing the percent of CNS mononuclear cells that are CD45^lo^ and CD45^hi/int^, F4/80+ macrophages. Data are shown as mean +/− SEM, n = 4 mice pooled/replicate, 4 replicates/experimental group. P values calculated using one-way ANOVA. C. (Top) Bar graph depicting the number of Iba-1^+^ cells within the cerebellum of vehicle treated or dexa treated MCMV infected mice. Data are shown as mean +/− SEM. The number of Iba-1^+^ cells was quantified from 4 sections/animal, n = 5–8 mice/experimental group. P values calculated using two-way ANOVA. (Bottom) Representative Iba-1 staining depicting activated macrophages within the cerebellum of vehicle treated or dexa treated infected mice, PND8, 20× scale bars = 50 µm. (Left) Iba-1-red, TOPROIII-blue, (Right) Black and white rendering of immunofluorescent images to increase contrast, white signals represent Iba-1 staining. D. Quantitative real-time PCR analysis of transcription of IFIT1, IFIT2 and STAT1 in the cerebellum of infected mice following treatment with dexa. Data are shown as mean +/− SEM, fold change normalized to control = 1, n = 5 mice/experimental group. P values calculated using two-way ANOVA.

Thus far our findings suggested that soluble factors produced by the inflammatory response to virus infection in the CNS were responsible for the global alterations in cerebellar development. Endogenous glucocorticoids have been demonstrated to protect against immune-mediated pathology in MCMV infected adult mice, suggesting that treatment with glucocorticoids could alter the pathological changes in the CNS of MCMV infected newborn mice [66], [67]. To examine the effects of glucocorticoid treatment on postnatal cerebellar development, control and MCMV infected mice were treated with dexamethasone (dexa), a glucocorticoid with potent anti-inflammatory activity, which has been routinely used in the treatment of CNS inflammation in both clinical medicine and experimental animal models of human disease [17], [68]–[72]. Control and MCMV infected newborn mice were treated daily with dexa or vehicle on PND4-6 and liver, spleen, brain and cerebellum were isolated from all groups on PND8. There was no significant difference in the number of plaque forming units (PFU) of virus in the spleen, liver or brain of dexa treated/infected animals when compared to vehicle treated/infected animals, signifying that treatment with dexa had minimal effects on viral replication (Figure 3A). We next assessed whether dexa treatment exhibited an anti-inflammatory effect following MCMV infection. Dexamethasone significantly reduced the frequency of CD45^hi/int^F4/80^+^ macrophages in the brains of infected mice compared to vehicle treated/infected mice (Figure 3B). Interestingly, the frequency of CD45^lo^ F4/80^+^ cells was reduced in the brains of MCMV infected mice as compared to control and dexa treated mice suggesting that the number of quiescent, or resting, microglia was decreased in infected animals, perhaps secondary to an increase in activated microglia in this experimental group ( Figure 3B). A reduction of MHC class II expression in this population was also observed in dexa treated/infected mice (data not shown). Similarly, the number of Iba-1^+^ cells was significantly decreased in the cerebellum of dexa treated/infected mice compared to vehicle treated/infected mice (Figure 3C). Consistent with the findings described above, the expression of IFIT1 was significantly decreased in the cerebellum of infected animals following treatment with dexa (Figure 3D). We also determined that dexa treatment normalized the expression of IFIT2 and STAT1 in the cerebellum of MCMV infected mice (Figure 3D). Together, these results demonstrated that dexa treatment decreased inflammation in the CNS of MCMV infected animals without significantly altering levels of virus replication.

### Treatment of MCMV infected mice with dexamethasone normalizes cerebellar development, but has significant off-target effects

**Figure 4:**
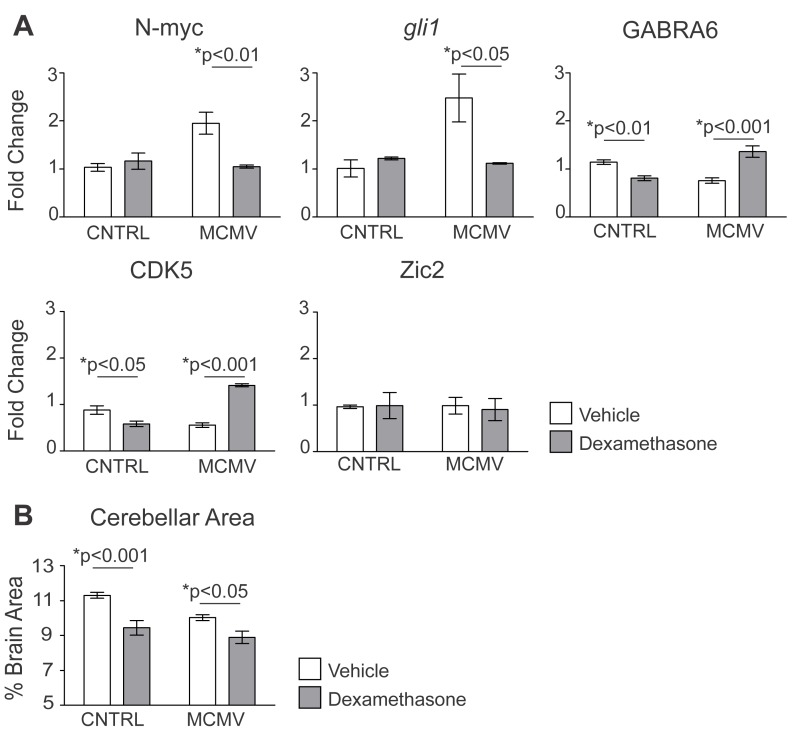
Treatment with the glucocorticoid dexamethasone normalizes developmental gene expression in the brains of infected mice but leads to cerebellar hypoplasia. A. Cerebellar expression of developmentally regulated genes from uninfected and infected mice treated with vehicle or dexa analyzed by quantitative real-time PCR. Data are shown as mean +/− SEM, fold change normalized to control = 1, n = 5 mice/experimental group. P values calculated using two-way ANOVA. B. Quantification of cerebellar area (expressed as a percentage of total brain area) in mice treated with vehicle or dexa. Data are shown as mean +/− SEM, measurements were taken from 5 sections/mouse, n = 5–7 mice/experimental group. P values were calculated by two-way ANOVA.

The finding that dexa treatment of MCMV infected mice significantly reduced the inflammatory response in the CNS raised the possibility that dexa treatment could also prevent the aberrant cerebellar development observed following infection. Dexamethasone treatment of infected mice normalized the expression of the developmentally regulated genes *gli1* and N-myc (both effectors of the sonic hedgehog (SHH) pathway responsible for granule neuron proliferation), as well as GABRA6 (a marker for granule neuron differentiation) and CDK5 (primarily expressed in differentiated neurons) (Figure 4A) [73]–[75]. Notably, some of these genes have previously been shown to be altered following MCMV infection [53]. However, dexa treatment of control animals also resulted in a significant reduction in the expression of both GABRA6 and CDK5 in the cerebellum when compared to control animals receiving only vehicle (Figure 4A). These differences in expression were not due to an effect of dexa on transcription because the expression of Zic2, a transcription factor expressed predominantly in granule neuron progenitors, was unaltered following treatment (Figure 4A) [76]. Morphometric measurements from the cerebella of infected mice demonstrated that the increased thickness of the EGL, previously associated with delayed migration of granule neuron progenitors, appeared to have been normalized following treatment with dexa (data not shown). However, the EGL in dexa treated/control animals was decreased in thickness compared to vehicle treated/control animals (data not shown). Dexamethasone treatment of control mice also resulted in a significant decrease in cerebellar area when compared to vehicle treated/control mice (Figure 4B). In addition, the cerebellar area of dexa treated/infected animals was further decreased compared to vehicle treated/infected animals (Figure 4B). Importantly, we did not observe a significant increase in activated caspase 3 staining in sections from these mice, indicating that increased apoptosis of granule neuron progenitor cells (GNPCs) did not contribute to the reduced size of the cerebellum in dexa treated/infected animals (data not shown). These findings suggested that dexa treatment of MCMV infected mice resulted in significant off-target effects in cerebellar development, a result that would limit the interpretation of findings from our studies of cerebellar development in dexa treated animals. Similar off-targets effects of dexa on cerebellar development have been previously described and thought to be secondary to the anti-proliferative effects of this specific glucocorticoid on GNPCs [77], [78]. Finally, our findings raised the possibility of an additive effect of dexa and MCMV infection on cerebellar development.

### Treatment of MCMV infected mice with prednisolone normalizes altered cerebellar development

**Figure 5:**
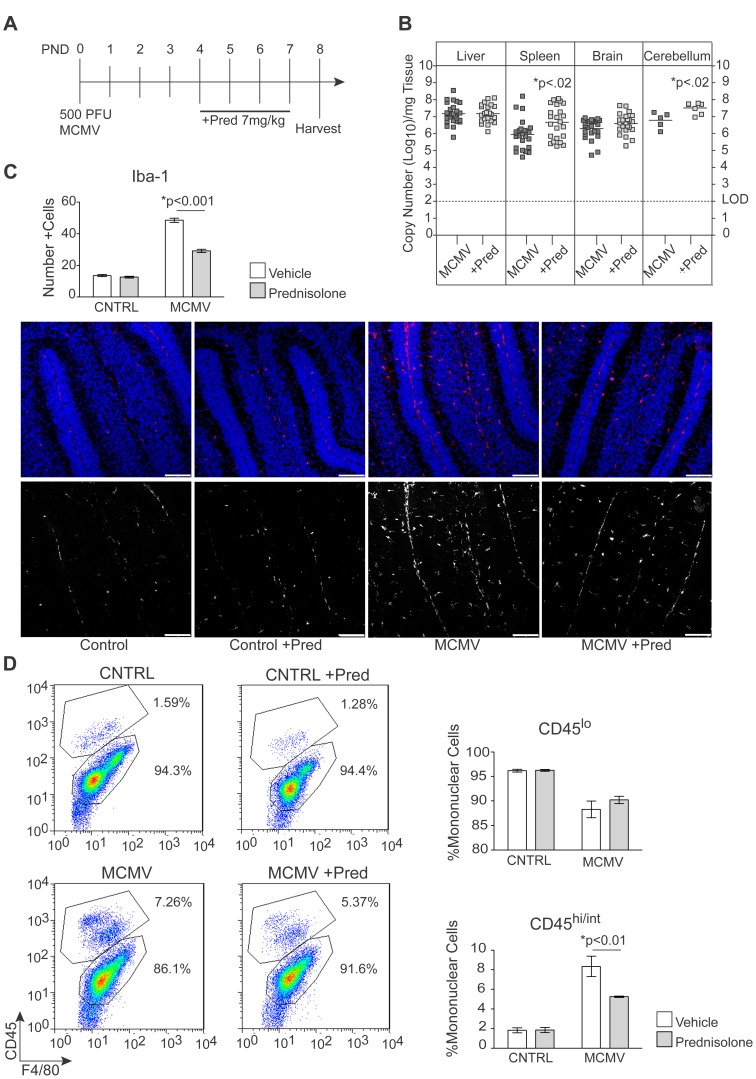
Treatment with prednisolone decreases the infiltration of inflammatory cells into the brain of MCMV infected mice. A. Schematic showing the time course of MCMV infection and prednisolone (pred) treatment. B. Quantitative real-time PCR for IE-1 showing viral genome copy number in the liver, spleen, brain and cerebellum of infected mice treated with vehicle or pred. P values calculated by Mann-Whitney test, n = 22–25 mice/experimental group for liver, spleen and brain; n = 5–7 mice/experimental group for cerebellum. C. (Top) Quantification of Iba-1+ cells in the cerebellum of vehicle or pred treated, control and infected mice. Data shown as mean +/− SEM. P values calculated by two-way ANOVA. Iba-1^+^ cells were counted in 4 sections/animal, n = 6 mice/experimental group. (Bottom) Panels of representative sections analyzed by confocal microscopy showing Iba-1 staining (red) and TOPROIII (blue) in the cerebellum of control or infected mice treated with vehicle or pred. Panel below represents black and white rendering of immunofluorescent images to increase contrast, white signals represent Iba-1^+^ cells. All images taken at 20×, scale bars = 50 µm. D (Left) Representative dot plots showing the percentage of CD45^lo^ and CD45^hi/int^, F4/80^+^ macrophages in the brain of vehicle or pred treated animals, gated on mononuclear cells. Plots are representative of 1 of at least 3 replicates, n = 4 mice pooled/replicate. (Right) Bar graphs showing the percentage of mononuclear cells that are CD45^lo^ and CD45^hi/int^, F4/80^+^ macrophages in the CNS. Data are shown as mean +/− SEM, n = 4 mice pooled/replicate, 3–5 replicates/experimental group. P values were calculated using two-way ANOVA.

The off-target effects of dexa on cerebellar development have been attributed to the resistance of this glucocorticoid to inactivation by 11β-hydroxysteroid dehydrogenase type 2 (11β-HSD2), an enzyme which is highly expressed in the postnatal cerebellum in rodents as well as humans [78]–[80]. This enzyme is induced by SHH during development of GNPCs in the cerebellar cortex and appears to be protective in terms of limiting both the apoptotic and anti-proliferative effects of corticosteroids [78], [79], [81], [82]. In contrast to dexa, other glucocorticoids such as hydrocortisone and prednisolone can be inactivated by 11β-HSD2 and have not been associated with the level of off-target effects observed following treatment with dexa [78]. Thus, we repeated the previous experiments using prednisolone (pred), a corticosteroid with predominant glucocorticoid activity, which has also been used to attenuate inflammation associated with infections of the CNS, both in animal models and clinical medicine [69], [83]–[86]. Control and MCMV infected newborn mice were treated once a day on PND4-7 with vehicle or pred. This time course of treatment was necessary secondary to the shorter in-vivo half-life of pred compared to dexa (Figure 5A) [87], [88]. Initially, we determined the effects of pred treatment on virus replication in MCMV infected mice. We found no significant difference between the level of virus replication in the liver or brain of pred treated animals compared to vehicle treated/infected animals. However, minimal increases in viral genome copy number were observed in both the spleen and cerebellum of pred treated/infected animals (Figure 5B).

We next determined the effect of pred treatment on the frequency of Iba-1^+^ cells in the cerebellum of both uninfected and MCMV infected mice. As described previously, the number of Iba-1^+^ cells was increased in the cerebellum of MCMV infected mice compared to control mice (Figure 5C). Following pred treatment, the frequency of Iba-1^+^ cells was reduced in MCMV infected animals (59% reduction) compared to vehicle treated/infected animals (Figure 5C). The number of Iba-1^+^ cells in pred treated/control animals was not significantly different from the number of positive cells in the cerebellum of vehicle treated/control animals (Figure 5C). The observed reduction of Iba-1^+^ cells in the cerebellum of pred treated/infected mice indicated that pred decreased macrophage/microglia activation in the CNS of newborn mice following MCMV infection. In agreement with our previous findings, treatment of infected mice with pred also decreased the frequency of CD45^hi/int^F4/80^+^ macrophages in the CNS of infected mice (Figure 5D). Treatment of control animals with pred had no significant effect on either the CD45^hi/int^F4/80^+^ macrophage population or the CD45^lo^ F4/80^+^ resting microglial population (Figure 5D). The observed reduction of Iba-1^+^ cells in the cerebellum and the decreased percentage of CD45^hi/int^F4/80^+^ macrophages in the CNS of pred treated/infected mice indicated that pred decreased the number of activated macrophage/microglia in the CNS of newborn mice following MCMV infection.

**Figure 6:**
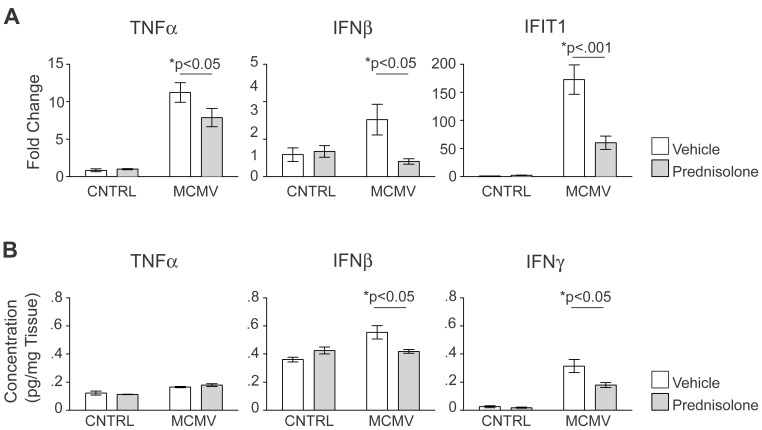
Treatment of infected neonatal mice with prednisolone decreases the expression of proinflammatory cytokines and interferon stimulated genes in the brain of MCMV infected mice. A. Quantitative real-time PCR analyzing the transcription of TNFα, IFNβ and IFIT1 in the cerebellum. Fold changed normalized to control = 1. Data shown as mean +/− SEM. P values calculated by two-way ANOVA, n = 5 mice/experimental group. B. Concentration of TNFα, IFNβ and IFNγ in the cerebellum of PND8 control and infected mice treated with vehicle or pred. Data are shown as mean +/− SEM, n = 3 cerebella pooled/replicate, 3 replicates/experimental group. P values were calculated using two-way ANOVA.

We next determined the effects of pred treatment on the expression of proinflammatory cytokines previously shown to be elevated in the cerebellum following MCMV infection (Figure 2D). Consistent with the findings described above, we observed a reduction in the transcription of TNFα (25%), IFNβ (70%) and IFIT1 (65%) within the cerebellum of MCMV infected mice treated with pred (Figure 6A). Pred treatment also decreased cytokine levels of IFNβ (25%) and IFNγ (43%) within the cerebellum (Figure 6B). Interestingly, cytokine levels of TNFα were not affected following pred treatment. These results illustrated that treatment with pred could attenuate MCMV induced inflammation in the CNS independent of changes in virus replication, thereby uncoupling the level of virus replication and the host inflammatory response within the cerebellum.

### Prednisolone treatment ameliorates inflammation associated cerebellar morphogenic abnormalities in MCMV infected mice

**Figure 7:**
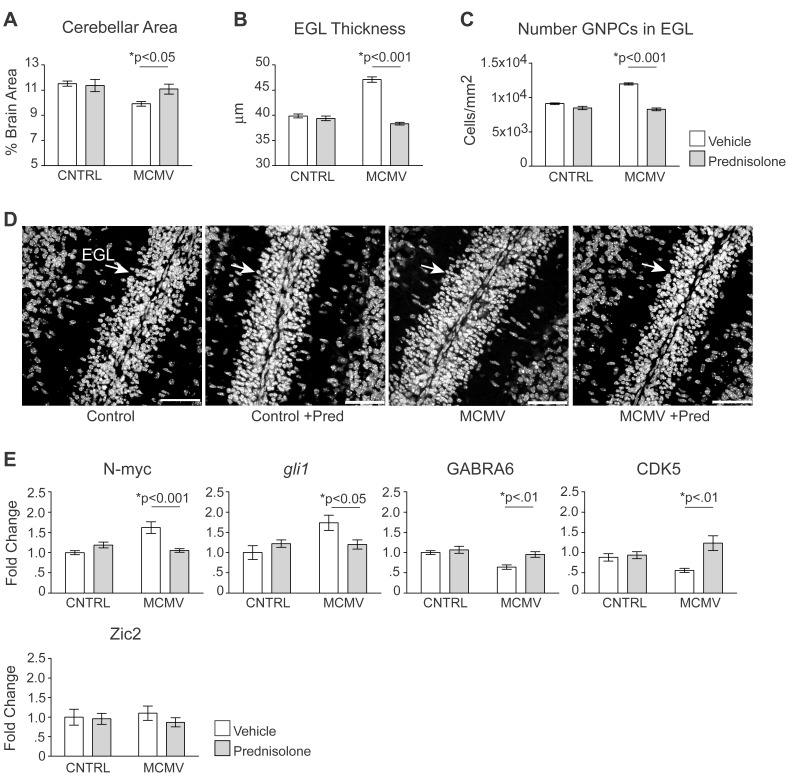
Treatment with the glucocorticoid prednisolone limits altered morphogenesis and developmental gene expression in the cerebella of MCMV infected mice. A. Cerebellar area of control or MCMV infected mice treated with vehicle or pred. Data are shown as mean +/− SEM. Stereological measurements from 5 sections/mouse, n = 5 mice/experimental group. P values calculated using two-way ANOVA. B. External granule cell layer (EGL) thickness in vehicle or pred treated control and MCMV infected mice. Data are shown as mean +/− SEM. EGL thickness was determined from 4 measurements/section, 8 sections/mouse, n = 6–8 mice/experimental group. P values calculated using two-way ANOVA. C. Granule neuron progenitor cells (GNPCs) in the cerebella of control and infected mice treated with vehicle or pred. Data are shown as mean +/− SEM. GNPC numbers from 8 sections were counted per mouse, n = 5–6 mice/experimental group. P values calculated using two-way ANOVA. D. Representative cerebellar sections showing a thickening of the EGL following MCMV infection that is corrected with pred treatment, 60×, scale bars = 20 µm. EGL containing GNPCs are shown in white, the adjacent molecular layer is shown in black. E. Transcription of developmentally regulated genes within the cerebellum of vehicle or pred treated, control and infected mice. Data are shown as mean +/− SEM. Fold change normalized to control = 1, n = 5 mice/experimental group. P values calculated using two-way ANOVA.

Since treatment with pred significantly reduced the inflammatory response in the CNS and has been reported to lack the off-target effects observed with dexa, we next determined if pred treatment could also limit the abnormal development of the cerebellum that was observed in MCMV infected animals. Because of the large number of mice used in these experiments, the variation in animal size and the size dependent variation in brain area, we normalized measurements of cerebellar area between experimental groups by expressing cerebellar area as a percentage of brain area. The ratio of cerebellar area/brain area was found to be similar for pred treated/control and pred treated/infected animals when compared to vehicle treated/control animals; however, vehicle treated MCMV infected mice showed a significant reduction in this ratio (Figure 7A). These results confirmed the decrease in cerebellar area previously observed following infection with MCMV and, more importantly, demonstrated normalization of altered cerebellar size in infected mice by treatment with pred. These findings were consistent with our hypothesis that inflammatory mediators, released in response to MCMV infection, were a primary cause of altered cerebellar development.

In addition to the decrease in cerebellar area, we have previously documented an increase in the thickness of the EGL in MCMV infected animals [53]. Since treatment of infected mice with pred lead to normalization of cerebellar area, we next determined whether this treatment would also normalize the increased thickness of the EGL following infection. As expected, the EGL was thicker in MCMV infected mice compared to control mice. This abnormality in cerebellar development was corrected in infected mice following treatment with pred (Figure 7B, D). There was no measureable difference in the thickness of the EGL in control animals treated with pred compared to vehicle treated/control animals (Figure 7B, D). To determine if the increase in the thickness of the EGL following infection was secondary to an increase in cellularity, the number of GNPCs in the EGL was quantified. Consistent with an increase in thickness, we found an increase in the number of GNPCs within the EGL following infection (Figure 7C). Concomitant with normalizing the increased thickness of the EGL, treatment of infected mice with pred also normalized the number of GNPCs within the EGL. We did not find any significant difference in the number of GNPCs in the EGL between vehicle treated/control animals or pred treated/control animals (Figure 7C). The normalization of MCMV induced abnormalities in the morphogenesis of the cerebellar cortex following treatment with pred demonstrated that we could limit morphogenic abnormalities within the cerebellum of infected mice by modulating inflammatory responses.

### Prednisolone attenuates inflammation associated impairments in cerebellar granule neuron precursor differentiation

Previously, we documented that following infection, morphological deficits within the cerebellum coincided with a significant reduction in the transcription of developmentally regulated genes expressed within GNPCs [53]. Since pred treatment reduced inflammation and corrected morphological deficits within the cerebellum of infected mice, we hypothesized that pred treatment could also correct abnormalities in the transcription of these genes. Similar to our studies using dexa, we assayed *gli1*, N-myc, GABRA6 and CDK5 expression in the cerebella of uninfected and MCMV infected mice treated with vehicle or pred. Consistent with our previous findings, expression of both GABRA6 and CDK5 was decreased following infection with MCMV when compared to control mice (Figure 7E). Following treatment with pred the expression of both genes was normalized within the cerebella of MCMV infected mice. Similarly, the transcription of *gli1* and N-myc was elevated in the cerebellum following infection and treatment of infected mice with pred decreased the expression of both genes (Figure 7E). Importantly, pred treatment had no effect on the transcription of *gli1*, N-myc, GABRA6 or CDK5 in control animals (Figure 7E). As a control, the expression of Zic2 was analyzed and was found to be similar in the cerebella of all groups (Figure 7E) [76]. These results indicated that decreasing inflammation in MCMV infected animals by treatment with pred normalized the expression of developmentally regulated genes in the absence of measurable off-target effects.

### Altered granule neuron proliferation following MCMV is corrected subsequent to treatment with prednisolone

**Figure 8:**
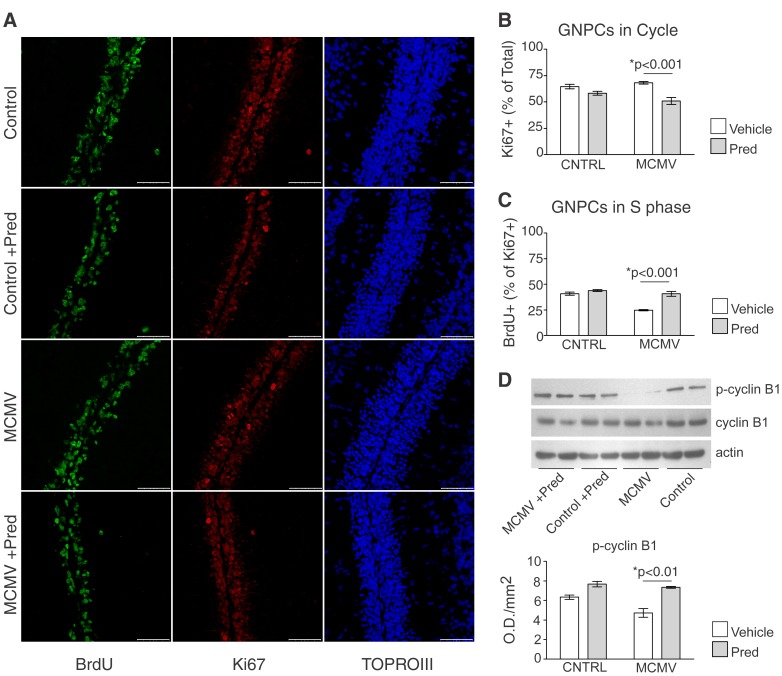
Treatment with the glucocorticoid prednisolone normalizes granule neuron progenitor cell proliferation in MCMV infected mice. A. Representative images of brain sections depicting the expression of cell cycle markers in the EGL of control or infected mice treated with vehicle or pred; BrdU (green), Ki67 (red), TOPROIII (blue), 60×, scale bars = 20 µm. B–C. Stereological quantification of BrdU^+^ and Ki67^+^ GNPCs in the EGL of vehicle or pred treated, control and infected mice. Data are shown as mean +/− SEM, 8 sections were counted per mouse, n = 5–6 mice/experimental group. P values calculated using two-way ANOVA. Vehicle treated control vs. MCMV were significantly different (p≤.001) as determined by two-tailed T test. D. (Top) Detection of phospho-cyclin B1 and cyclin B1 in the cerebellum by immunoblotting. Actin loading control shown at bottom. Each lane represents 2 cerebella pooled, n = 2 lanes/experimental group. (Bottom) Densitometry showing the expression of p-cyclin B1, relative to actin, in the cerebellum of vehicle or pred treated, control and infected animals. Data are representative of 3 replicate blots. P value calculated by two-way ANOVA. Control vs. MCMV were significantly different (p≤.02) as determine by two-tailed T test.

In MCMV infected mice, the upregulation of *gli1* and N-myc was inconsistent with the deficit in GNPC proliferation observed in our previous studies [53]. This suggests that an alternative mechanism could be responsible for the deficit in GNPC proliferation within the cerebellum of infected mice [53]. Given our previous findings (increased thickness of the EGL, decreased GNPC differentiation, decreased GNPC migration to the IGL and decreased thickness of the IGL), we postulated that a block or delay within the GNPC cell cycle, downstream from the actions of *gli1* and N-myc, would be most consistent with our observations. The failure of GNPCs to complete a program of proliferation in the EGL would prevent their differentiation and subsequent migration into the IGL. This mechanism would also account for the increased cellularity of the EGL and the decreased cellularity of the IGL in infected animals [53], [89]–[91]. To investigate this possibility, PND8 animals were injected with BrdU, a marker of cells in S phase. Serial sections from the cerebellum were stained with antibodies reactive with BrdU and Ki67, a marker of cycling cells, and the number of positive cells was quantified for each marker (Figure 8A). No difference was observed in the percent of total GNPCs that were positive for Ki67 in the EGL of MCMV infected animals compared to control animals (Figure 8B). However, a decrease in the percent of cycling cells (Ki67^+^) positive for BrdU was detected in infected animals when compared to control animals (Figure 8C). The decrease in BrdU reactivity within GNPCs of infected mice was therefore not secondary to a decrease in the overall number of cells in the cell cycle. Moreover, the previously described minimal level of apoptosis of GNPCs in either group of animals indicated that there is likely a block or delay in the cell cycle of GNPCs following infection [53].

If the inflammatory response in the CNS of infected mice contributed to the block/delay in the proliferation of GNPCs, our results described above would argue that the anti-inflammatory effects of pred could alleviate this block and restore the proliferative capacity of GNPCs in the EGL. Analysis of Ki67 expression in pred treated groups revealed that the percent of GNPCs in the cell cycle was similar to that of infected or control animals that were treated with vehicle (Figure 8B). When compared to vehicle treated/control animals there was no significant difference in the percent of BrdU^+^ cells in EGL of pred treated/infected animals indicating that pred treatment of infected animals normalized the deficit in GNPC proliferation associated with MCMV infection (Figure 8C). Importantly, the percent of BrdU^+^ GNPCs in the EGL of pred treated/control animals was not significantly different from vehicle treated/control animals. Together, these findings argue that pred treatment alleviated alterations in the cell cycle of GNPCs that were associated with MCMV infection. Furthermore, these results support our hypothesis that modulating the inflammatory response following MCMV infection could limit deficits in cerebellar morphogenesis, likely through reversing the delay in GNPC proliferation.

To further define the disruption in the cell-cycle of GNPCs following infection we assayed the levels of two cyclins, cyclin D1 and cyclin B1, in control and MCMV infected mice. Levels of cyclin D1 were not significantly different between control or infected animals, suggesting that infection with MCMV did not alter the signals associated with entry of GNPCs into G1 (data not shown) [92]. Similarly, pred treatment did not alter cyclin D1 levels in infected or control animals (data not shown). Although there was no observable difference in the levels of total cyclin B1 expression between infected and control mice (Figure 8D), the level of phosphorylated-cyclin (p-cyclin) B1, a marker for G_2_/M, was decreased within the cerebella of infected animals compared to control animals (Figure 8D) [93], [94]. Together with the decreased number of BrdU^+^ GNPCs, this data further argued for a block/delay in the cell cycle following infection. Cerebella from both control and MCMV infected mice treated with pred displayed levels of p-cyclin B1 that were similar to vehicle treated/control mice (Figure 8D). Although this data did not reveal the precise point where cell cycle progression was delayed, it further confirmed that altered development of the cerebellum in infected animals was associated with delayed proliferation of GNPCs within the EGL. Treatment with pred corrected this deficit and normalized the morphological abnormalities within the cerebellum following infection. These results were consistent with a mechanism in which the developmental abnormalities associated with focal encephalitis in MCMV infected newborn mice resulted from the host inflammatory response as opposed to a direct virus-mediated mechanism.

## Discussion

Previously we have shown that intraperitoneal inoculation of newborn mice with MCMV resulted in a focal CNS infection that involved all regions of the brain that but did not exhibit specific cellular tropism [53]. Histologically, the foci consisted of a small number of virus-infected cells, mononuclear cells and reactive astroglial cells [53]. Although there was no observable difference in the size of the cerebrum between infected and uninfected animals, cerebellar hypoplasia was readily apparent in infected animals and was associated with delayed foliation and decreased area of the cerebellar cortex, findings attributable to the decreased proliferation of GNPCs within the EGL [53]. Morphogenic abnormalities of the cerebellar cortex included increased thickness of the EGL, decreased thickness of the IGL, abnormal arborization of Purkinje neuron dendrites and thinning of the molecular layer [53]. Interestingly, the altered morphogenesis of the cerebellum was symmetric even though foci containing virus infected resident cells and infiltrating mononuclear cells were scattered widely throughout the parenchyma of the cerebellum. These later findings strongly argued that the developmental abnormalities were secondary to a soluble mediator generated during virus-induced inflammatory responses in the CNS and not from direct cytopathic effects of virus infection. In this report, we have described findings consistent with this mechanism; specifically, evidence that attenuation of inflammatory responses in infected mice, by treatment with anti-inflammatory glucocorticoids, normalized developmental abnormalities in the cerebellum without affecting the level of virus replication.

Our results demonstrated that several measures of GNPC proliferation were altered in MCMV infected mice, including a decrease in the frequency of cells in S phase and a decrease in the levels of phospho-cyclin B1 within the EGL of MCMV infected mice. Several explanations could account for these findings, including a decrease in the number of GNPCs entering the cell cycle, premature exit of GNPCs from the cell cycle and a block or delay in the cell cycle of GNPCs following infection. Premature exit of GNPCs from the cell cycle represented an obvious explanation for the decreased cerebellar size but other measures of GNPC proliferation were inconsistent with this explanation. The increased cellularity of the EGL following MCMV infection and the similar percentages of Ki67+ GNPCs in infected and control mice argued that there was no difference in the number of GNPCs entering the cell cycle nor was there an increased number of GNPCs exiting the cell cycle. Because we found a decrease in certain markers of proliferation but no change in the number of cycling GNPCs following MCMV infection in this study as well as in a previous study, a more consistent interpretation of our data is that the cell cycle of GNPCs in the EGL is prolonged in MCMV infected animals [53]. Prolongation of the cell cycle could delay the completion of the programmed proliferation and subsequent differentiation of GNPCs that is required for normal morphogenesis of the cerebellar cortex. Variation in the rate of cell division of GNPCs in the EGL has been described, suggesting that the duration of the cell cycle in these cells is not autonomous and can be influenced by extracellular cues [90], [95], [96]. Though we have not fully characterized the nature of this alteration in the cell cycle of GNPCs, it was reversible, in that the delay was corrected when MCMV infected animals were treated with glucocorticoids.

Although a unifying mechanism for the normalization of cerebellar development in pred treated MCMV infected mice remains incompletely described, our results were most consistent with a decrease in the inflammatory response in the CNS leading to normalization of the proliferative capacity of GNPCs in the cerebellar cortex. This mechanism is based on previous studies that have demonstrated that GNPCs undergo what is thought to be a programmed number of cell divisions prior to exiting the cell cycle, entering a differentiation program and then migrating from the EGL into deeper layers of the cerebellar cortex [90], [95], [96]. This well choreographed developmental pathway has been extensively studied and many of the molecular signals associated with this pathway have been described [74], [91], [95]–[100]. We are proposing that if the cell cycle of GNPCs is prolonged, subsequent to inflammation in the cerebellum, then normal morphogenesis of the cerebellar cortex fails to take place and the expression of developmentally regulated genes that depend on differentiation and correct cellular positioning will be delayed. Findings from this study are consistent with a reversible, generalized slowing of the GNPC cell cycle in infected mice. Reversal of this slowing could be expected to result in a rebound in GNPC proliferation, permitting the completion of the developmentally programmed cell divisions, differentiation into migrating granule neurons, migration into the IGL and expression of the associated differentiation genes. The reversibility of this mechanism is consistent with the partial resolution of defects in cerebellar development observed in vehicle treated MCMV infected mice following virus clearance and regulation of the inflammatory response later in infection [53]. Additional support for the reversibility of a slowing of the cell cycle has been reported in a study of 11β-HSD2 −/− transgenic mice treated with corticosterone [79]. Findings from this study demonstrated a rebound in the cerebellar area and the size of the IGL in these transgenic mice following withdrawal of steroid treatment [79]. Even though the effector molecules and pathways that lead to altered proliferation of GNPCs and cerebellar development in this model of a human CNS infection remain undefined, such a mechanism could argue for a common pathway leading to the developmental abnormalities associated with inflammation following infection of the developing brain of the fetus and newborn infant by a number of microbial agents. Alteration in the rate of proliferation of progenitor cells in the developing CNS could lead to deficits in developmental, stage dependent cell positioning and potentially result in a number of long term neurological abnormalities.

A recent study that carefully detailed the effects of glucocorticoids on the developing cerebellum described several phenotypes following treatment with different glucocorticoids [78]. These investigators demonstrated that the phenotypic response of GNPCs to glucocorticoids was dependent on the presence of 11β-HSD2, an enzyme that is expressed at higher levels in the cerebella of both newborn rodents and humans as compared to other regions of the CNS [78]–[80], [101]. Previous studies have indicated that the inactivation of glucocorticoids by 11β-HSD2 limits the anti-proliferative and apoptotic inducing activities of endogenous and exogenous glucocorticoids [78], [79]. Because dexamethasone (dexa) is not efficiently inactivated by 11β-HSD2, treatment of neonatal mice with dexa resulted in increased GNPC apoptosis (short term treatment) or decreased GNPC proliferation (chronic treatment), secondary to exit from the cell cycle presumably from accelerated GNPC differentiation [77], [78]. Interestingly, in this study chronic prednisolone (pred) treatment resulted in an intermediate phenotype due to the inactivation of this specific glucocorticoid by 11β-HSD2 [78]. Our findings were consistent with the results presented in this report in that treatment with dexa, but not pred, resulted in a significant decrease in the size of the cerebellar cortex in both uninfected and infected mice. We also noted that in two independent experiments the cerebellar area in dexa treated/infected mice was smaller than that of both dexa treated/control mice or vehicle treated MCMV infected mice. These findings suggested that the effects of dexa and MCMV infection were additive and raised the possibility that the effect of dexa on GNPC proliferation in this setting differed from those that followed MCMV infection. Interestingly, dexa treatment did result in normalization of the expression of genes associated with GNPC differentiation (GABRA6 and CDK5) in the absence of normalization of GNPC proliferation, a finding consistent with accelerated GNPC differentiation in animals following treatment with dexa [77], [78]. The premature exit of GNPCs from the cell cycle likely accounted for the cerebellar hypoplasia and decreased cerebellar area that was observed in dexa treated animals. In contrast, when infected mice were treated chronically with pred, we observed a correction of the abnormal cell cycle of GNPCs that was also associated with normalization of the morphogenic abnormalities in the cerebellar cortex. Following normalization of the cell cycle in pred treated animals, GNPCs completed their programmed proliferation in the EGL, migrated into the deeper layers of the cerebellum and expressed development specific genes. We have not identified a specific mechanism(s) to explain the correction of proliferation deficit(s) in GNPCs following pred treatment, but it is unlikely that in pred treated mice, GNPCs exited the cell cycle and differentiated as was observed in dexa treated mice. This argument is based on three findings; (i) a similar frequency of GNPCs were cycling in both pred treated and vehicle treated mice, (ii) the frequency of BrdU^+^ GNPCs in the EGL was increased following pred treatment and (iii) measures of cerebellar morphogenesis (EGL thickness, cerebella area and EGL cellularity) were normalized in infected mice following treatment with pred. Several experimental models of CNS infection in newborn animals have also noted beneficial outcomes following treatment with anti-inflammatory agents, but in some cases and in contrast to our findings, increased disease severity secondary to increased replication of the microorganism was also observed [17], [67], [68]. Experimental rodent models of herpes simplex encephalitis have demonstrated a beneficial effect of steroid treatment when combined with an antiviral agent suggesting that host-derived inflammation contributes to the outcome of CNS infection with this virus [102], [103]. In findings that paralleled our results, treatment of Borna disease virus (BDV) infected adult rats with dexa limited inflammation and also appeared to improve neurologic function in infected animals [17]. In clinical medicine, the use of glucocorticoids to limit CNS inflammation in patients with mycobacterial infections of the brain is well established [69], [70]. These agents have also been utilized to limit neurological sequelae that follow bacterial meninigitis associated with pyogenic bacteria [71]. Several studies have demonstrated that glucocorticoids efficiently limit the innate immune response to microorganisms in the CNS, including the expression of proinflammatory cytokines, chemokines and interferon stimulated genes [17], [104]. However, the use of glucocorticoids, particularly dexa, in young infants remains controversial because of the well documented adverse effects this agent has on brain development [105], [106].

The importance of SHH in the proliferation of GNPCs in the cerebellar cortex has been studied extensively [107]–[113]. The proliferation of these neuron progenitors in response to SHH has been reported to involve the transcription factors *gli1* and N-myc [109], [114]–[117]. It was therefore somewhat unexpected to find that expression of both *gli1* and N-myc was increased in the cerebella of MCMV infected mice as compared to control mice. Interestingly, we noted that transcription of *patched (Ptch)* was also increased in the cerebella of MCMV infected mice, a finding that paralleled the increased expression of *gli1* and could represent a regulatory response to SHH induced responses [118], [119]. We do not have a definitive explanation for the increase in *gli1* and N-myc expression but noted that when MCMV infected mice were treated with glucocorticoids the expression of these SHH effectors was normalized. Consistent with our observations, previous reports have suggested that proinflammatory cytokines can modulate the SHH pathway [120], [121]. As an example, increases in GNPC proliferation have been documented in transgenic mice with constitutive IFNγ expression in the CNS [122]. In these engineered mice, SHH and *gli1* expression was induced by IFNγ via a STAT1 dependent pathway. More recent studies have reported that IFNγ treatment of cultured granule neurons leads to increased proliferation and that STAT1 binds directly to the SHH promoter [123], [124]. Interestingly, both IFNγ and STAT1 were upregulated in the cerebella of MCMV infected mice coincident with an increase in the expression of N-myc and *gli1* (Figure 3D; Figure 6B). Moreover, treatment with pred reduced the cytokine levels of IFNγ and normalized the expression of both N-myc and *gli1* following MCMV infection. Studies of cytokines during CNS development have detailed both neuroprotective and deleterious roles, suggesting a delicate balance between the homeostatic and immune functions of cytokines in the developing CNS [125]–[128]. Our findings suggest that cytokines released following neonatal infection with MCMV could have deleterious effects on developing GNPCs within the cerebellum and that modulating the inflammatory response associated with this infection could limit damage to the developing CNS.

An important aspect of this study is that the pathological and histopathological findings in this murine model appear very similar to those reported in human infants with congenital CMV infection. The focal encephalitis, characteristic of MCMV infection in mice, has also been noted in autopsy findings from infants with congenital HCMV infections. Furthermore, in this model histopathological findings of mononuclear cell infiltrates and reactive gliosis, termed micronodular gliosis, are remarkably similar to those found in infected human infants [55], [57], [60], [129], [130]. Cerebellar hypoplasia is an invariant finding in this murine model and also frequently reported in infants with congenital HCMV infections that have been studied by imaging or, in a smaller number, following autopsy [57], [131], [132]. Reports describing MRI findings in infants with congenital HCMV infection have suggested that cerebellar hypoplasia is characteristic of this intrauterine infection. However, it should also be noted that the murine model we have developed has a significant limitation, dictated by the route of virus inoculation and the age of the developing brain at the time of infection. CNS development in newborn mice is believed to be at a stage similar to that of a mid to late 2nd trimester human fetus. Thus, in the murine model we have developed, cortical damage associated with an earlier gestational age of fetal infection will not be adequately modeled. Yet it is also important to note that the vast majority of infants with congenital HCMV infections also do not exhibit structural damage to the cerebral cortex, raising the possibility that only a minority of infants are infected early in gestation. In agreement with this possibility, recent studies have provided evidence suggesting that transmission of virus to the developing fetus occurs more frequently in the later stages of pregnancy [133]. Thus, with the awareness of limitations inherent in studies carried out in rodents, we would argue that the findings we have generated from our studies suggest that inflammation in the developing brain should be considered a potential contributor to at least some of the developmental abnormalities that have been associated with intrauterine HCMV infections. Furthermore, if inflammation and the soluble mediators present in the CNS account for the altered proliferative capacity of neural progenitor cells, our results could be extrapolated as a potential explanation for maldevelopment of the brain associated with other intrauterine infections resulting in CNS inflammation.

Even though our findings in this murine model of congenital CMV infection have demonstrated a beneficial effect of glucocorticoid therapy in maintaining the developmental program during MCMV infection, we cannot directly extrapolate our findings in this model system to human disease or other infections of the CNS. However, the potential intersections between neurodevelopmental pathways and those that contribute to CNS inflammation in neonatal animals would suggest that more selective approaches to limiting CNS inflammation could open new therapeutic avenues and lead to improved outcomes. These approaches combined with antiviral therapy, to limit virus replication until host responses can efficiently clear virus from the CNS, could offer a more optimal approach for management of this important perinatal infection. Further exploitation of this model could provide insight into the feasibility of such an approach and perhaps aide in defining markers of CNS inflammation, allowing for a more selective introduction of anti-inflammatory therapy.

## Materials and Methods

### Ethics statement

All animal breeding and experiments were performed in accordance to the guidelines of the University of Alabama – Birmingham Institutional Animal Care and Use Committee (IACUC) in strict compliance with guidelines set forth by the NIH (OLAW Assurance Number - A3255-01). Research was conducted under a protocol approved by IACUC. All experiments done at the University of Rijeka were in accordance with the University of Rijeka – Croatia animal use and care policies in accordance to the guidelines of the animal experimentation law (SR 455.163; TVV) of the Swiss Federal Government.

### MCMV infection and corticosteroid treatment

Infection of mouse pups was performed as previously described [53]. Briefly, newborn Balb/c mice (6–18 hrs post-partum) were infected with 500 PFU of MCMV-Smith (ATCC VR-1399) by i.p. (intraperitoneal) inoculation. Control and MCMV infected pups were treated on PND4-6 by i.p. injection with dexamethasone sodium phosphate (dexa; APP Pharmaceuticals); 1 mg/kg in 50 µl of sterile PBS. Dexa was administered once a day and mice were sacrificed on PND8 between 36 and 42 hours after the last treatment was administered. For Prednisolone experiments, animals were treated with prednisolone sodium phosphate (pred; commercial pharmacy); 7 mg/kg (equivalent to 1 mg/kg dexa) in 50 µl of sterile PBS on PND4-7. Treatments were administered once a day and mice were sacrificed on PND8 between 16 and 18 hrs post injection. As a control, uninfected and MCMV infected animals were given i.p. injections with 50 µl sterile PBS alone (vehicle). Animals were sacrificed on PND8, perfused with ice cold PBS and organs were harvested and processed for the appropriate downstream application. All mice were purchased from The Jackson Laboratory (Bar Harbor, ME).

### Virus growth and titer analysis

Stocks of MCMV-Smith strain were propagated by infection of mouse embryonic fibroblasts (MEFs). Infected media was harvested at 5–7 days post-infection and frozen at −80°C. For dexa experiments, organs were collected, weighed and homogenized. A 10% homogenate in media was utilized for standard plaque assays [134]. For pred experiments, organs were collected and DNA was isolated using Trizol according to the manufacturer's instructions (Roche Applied Science). 1 µg of DNA was then used for quantitative real-time PCR with the following primers for MCMV IE-1 Exon 4: *Forward:* 5′-*GGC TTC ATG ATC CAC CCT GTT A* – 3′; *Reverse:* 5′-*GCC TTC ATC TGC TGC CAT ACT* – 3′. Primers were used at a concentration of 250 nM/reaction. The following FAM-TAMRA (BHQ-2) probe was used at a concentration of 300 nM/reaction for real-time detection: 5′-/56-FAM/ *AGC CTT TCC TGG ATG CCA GGT CTC A* – 3′. Real time PCR was performed by Taqman based assay using the StepOne Plus system from Applied Biosystems (Carlsbad, CA).

### Immunofluorescence, immunohistochemistry and cerebellar morphometry

For immunofluorescence studies, mice were injected on PND8 with 50 µg/g of BrdU (Sigma Aldrich) in 1× PBS, 6 hrs. prior to harvest. Mice were then perfused with PBS and brains were fixed in 4% paraformaldehyde (PFA) overnight, cryoprotected in 30% sucrose-PBS and embedded in Tissue Tek O.C.T. compound (Andwin Scientific). 8-µm sagittal sections were cut using a Leica cryostat. Cut sections were dried for 4 hours at room temperature (RT), rehydrated in 1× PBS then used for immunofluorescence assays. For Iba-1 staining, sections were blocked in 1× PBS, .05% Triton X-100, 20% normal goat serum, 5% BSA for 2 hr. at RT. Sections were then stained with anti-Iba-1 overnight at 4°C. Subsequently, sections were washed with PBS, .05% Triton X-100 and then incubated for 2 hrs. at RT in the dark with secondary antibody, followed by a 15 min. incubation with TOPRO-3 iodide (1∶1000, Molecular Probes) at RT. Following staining for Iba-1, sections were post-fixed with 2% PFA for 20 min. at RT. Sections were washed and mounted using Vectashield Fluorescent mounting medium (Vector Laboratories). For BrdU/Ki67, sections were blocked in 1× PBS, 1% Triton X-100, 20% normal goat serum, 1 M glycine, 5% BSA for 1 hr. at RT. Blocking was followed by a 2 N HCL acid wash for 10 min. on ice, 10 min. at RT and 20 min at 37°C. Sections were then buffered in .1 M Borate buffer for 12 min. at RT, washed in PBS, 1% Triton X-100 and labeled as previously described. Primary antibodies utilized in this study were anti-Iba-1 (1∶200, Wako, Japan), anti-Ki67 (1∶200, ab66155; Abcam), anti-IE1 (Chroma101 [53]) and anti-BrdU (1∶50, ab6326; Abcam). Secondary antibodies used were: Alexa Fluor 594 - conjugated anti-Rabbit; Alexa Fluor 488 - conjugated anti-mouse (Molecular Probes) and Goat anti-Rat – FITC (Southern Biotech), respectively. Images of stained sections were collected by using an Olympus Fluoview confocal microscope (20× objective for Iba-1 and 60× objective for BrdU/Ki67). For cell counts, images were saved as TIFF files and opened in Image J [135]. An area box was created and the number of cells in the EGL within this box was counted for each section.

Frozen sections were used for all morphometric measurements. EGL measurements were done on serial sections using Image J software. Measurements were obtained from sections stained with BrdU, Ki67 and TOPRO3. Images were collected with a confocal microscope. 4 measurements were taken from the primary fissure of the EGL in each section and 8 serial sections were measured per animal. For area measurements, the first 5 sections in each series were stained with 1% cresyl violet in ethanol for 10 min. followed by washing with 1× PBS until dye no longer ran off. Sections were mounted with 50% glycerol, 50% PBS and pictures were taken using an Olympus BX41 microscope with a 2× objective. Representative sections showing a close up of the cerebellum used in the paper were obtained with a 4× objective. Cerebellar area and brain area was measured using Image J software [135].

### Flow cytometry

CNS mononuclear cells were isolated by using a percoll density gradient protocol [62]. Isolated cell populations were stained in FACS buffer (2% BSA and 0.2% sodium azide) for 30 min at 4°C in the dark and fixed in 2% PFA. All samples were stained with CD45-FITC and F4/80-APC (eBioscience) and MHCII-IA/IE (Biolegend). Samples were acquired using a FACSCalibur (BD Biosciences) flow cytometer and analyzed using FlowJo7.6.1.

Due to low cell number and poor cell viability, mononuclear cell isolations from neonatal brain was performed as follows for prednisolone treated groups. Brains were homogenized using a GentleMACs tissue homogenizer (Milteniy Biotech). Homogenates were strained through a 40 µm nylon strainer, followed by centrifugation at 400×g for 4 min at 4°C. Homogenates were washed once with 1×PBS (without Ca^++^/Mg^++^) and centrifuged again at 400×g, 4 min at 4°C. Mononuclear cells were isolated by resuspending the pellet in a 37% continuous Percoll gradient followed by centrifugation at 690×g for 20 min, 4°C with gentle braking. Pellets were washed once with FACS buffer (1×PBS, 2% BSA, .2% Sodium Azide), then lysed for 5 min with 1 ml RBC lysis buffer (Sigma Aldrich). Lysis was inhibited by adding 10 mls FACS buffer and the pellet was collected by centrifugation (400×g, 4 min at 4°C). Pellets were again washed with FACS buffer, followed by resuspension in FACS buffer with FC block (1∶100, eBioscience). Mononuclear cells were blocked for 30 min on ice, counted using a TC20 cell counter (Bio-Rad) and 100 µl of cell suspension was transferred to individual wells of a round bottom, polystyrene 96 well plate. 100 µl of FACS buffer was added to each well and the plate was centrifuged (400×g, 4 min at 4°C) to pellet the cells. Mononuclear pellets were washed 2× with FACS buffer, followed by staining with CD45 – PerCP (1∶300), Cd11b – PE (1∶200) and F480 – FITC (1∶300) (eBioscience) for 1 hr at 4°C in the dark. Following staining, 150 µl of FACS buffer was added to each well and cells were pelleted by centrifugation. Cells were again washed 2× with FACS buffer followed by fixation with 4% PFA for 20 min at 4°C in the dark. Following fixation, cells were washed 2× with FACS buffer, resuspended in 200 µl FACS buffer and transferred to 5 ml polystyrene FACS tubes (BD Falcon). Samples were acquired using a FACSCalibur (BD Biosciences) flow cytometer and analyzed using FlowJo7.6.1. Dexamethasone experiments were repeated using this protocol and data were compared to the previous protocol. No differences were observed in the frequency of CD45^lo^ or CD45^hi/int^/F480^+^ mononuclear cell populations in any group when compared to our previous findings; however, mononuclear cell numbers were greatly improved.

### Quantitative real time PCR

Total cerebellar RNA from control and experimental mice was isolated using Trizol reagent (Roche Applied Science); 500 µl Trizol/cerebellum according to manufacturer's protocol. cDNA from each sample was synthesized using the Superscript III First Strand synthesis kit (Invitrogen). Taqman based real time PCR was employed for determining the mRNA expression of genes of interest in experimental animals relative to uninfected controls. Taqman assay mixes for TNF-α (Mm99999068), IFN-β (Mm00439552), STAT1 (Mm00439518), IFN-γ (Mm99999071), *gli1* (Mm00494645), N-myc (Mm00476449), Zic2 (Mm01226725), CDK5 (Mm00432437) and GABRA6 (Mm01227754) were obtained from Applied Biosystems. Real time PCR was performed using the StepOne Plus system from Applied Biosystems. The housekeeping gene 18S was used as a control for all experiments. The fold change (target gene expression relative to 18S) for control animals was set to a value of 1 +/− SEM and the relative fold change for each experimental group was determined by normalizing to control animals.

### ELISA

Cerebella were harvest from PND8 animals. Samples were pooled (3 cerebella/sample) and homogenized in ELISA buffer (1×PBS, .25% Triton X-100) containing protease/phosphatase inhibitors (Thermo Scientific). Lysates were rotated for 20 min at 4°C then sonicated 3× for 5 sec, followed by centrifugation at 12K× g for 10 min at 4°C. Aliquots were made and stored at −80°C until use. ELISAs were performed according to the manufacturer's instructions: TNFα (eBioscience), high sensitivity IFNγ (ebioscience, San Diego, CA) and IFNβ (PBL Interferon Source). Cytokine concentrations (pg/ml) were normalized for amount of tissue used (mg).

### Immunoblot of cerebellar lysates

Cerebella harvested from control and experimental groups at PND8 were homogenized in RIPA buffer (50 mM Tris-HCl, NaCl 150 mM, 1% NP-40, 0.25% Na-Deoxycholate, 1 mM EDTA) containing protease/phosphatase inhibitors (Thermo Scientific) and cleared of insoluble material by centrifugation at 12K× g. 50 µg of protein solubilized in sample buffer (5% SDS,2% 2-mercaptoethanol, Tris pH 8) and separated by SDS-PAGE electrophoresis using a 10% acrylamide gel. Electrophoretically separated proteins were immobilized on nitrocellulose membranes and used for Western blot analysis. Membranes were probed overnight at 4°C for actin (1∶1000, MAB1501; Millipore), cyclin D1, cyclin B1 and phospho-Cyclin B1 (Ser 147) (1∶500, 2978, 4138 and 4131 respectively; Cell Signaling Technology). Immunoblots were incubated for 1 hr with HRP-conjugated anti-mouse or anti-rabbit secondary antibodies (Southern Biotech) then developed with ECL reagent (Perkin Elmer). Densitometry was performed using Quantity One software (Bio-Rad) and levels of protein were normalized to actin for each lane.

### Statistics

Statistical significance of comparisons of mean values was assessed by a two-tailed Student's t test, one-way analysis of variance (ANOVA) followed by Bonfferronni's multiple comparison test, two-way ANOVA followed by Bonfferronni's posttest, or a Mann-Whitney test using Prism 4 software (GraphPad).
